# Targeting human milk fortification to improve very preterm infant growth and brain development: study protocol for Nourish, a single-center randomized, controlled clinical trial

**DOI:** 10.1186/s12887-021-02635-x

**Published:** 2021-04-09

**Authors:** Mandy B. Belfort, Lianne J. Woodward, Sara Cherkerzian, Hunter Pepin, Deirdre Ellard, Tina Steele, Christoph Fusch, P. Ellen Grant, Terrie E. Inder

**Affiliations:** 1 Department of Pediatric Newborn Medicine, Brigham and Women’s Hospital, 221 Longwood Avenue, BL-341, Boston, MA 02115 USA; 2 Harvard Medical School, Boston, MA USA; 3 School of Health Sciences and Child Wellbeing Research Institute, University of Canterbury, Christchurch, New Zealand; 4 Department of Nutrition, Brigham and Women’s Hospital, Boston, MA USA; 5 Department of Nursing, Brigham and Women’s Hospital, Boston, MA USA; 6 Department of Pediatrics, Paracelsus Medical School, Nuremberg, Germany; 7 Division of Newborn Medicine, Boston Children’s Hospital, Boston, MA USA

**Keywords:** Human milk, Nutrition, Very preterm infant, Neonatal intensive care, Growth, Neurodevelopment, Magnetic resonance imaging, Randomized trial

## Abstract

**Background:**

Human milk is recommended for very preterm infants, but its variable macronutrient content may contribute to undernutrition during a critical period in development. We hypothesize that individually targeted human milk fortification is more effective in meeting macronutrient requirements than the current standard of care.

**Methods:**

We designed a single-center randomized, controlled trial enrolling 130 infants born < 31 completed weeks’ gestation. Participants will receive fortified maternal and/or pasteurized donor milk but no formula. For participants in the intervention group, milk will be individually fortified with protein and fat modulars to achieve target levels based on daily point-of-care milk analysis with mid-infrared spectroscopy, in addition to standard fortification. The study diet will continue through 36 weeks’ postmenstrual age (PMA). Clinical staff and parents will be masked to study group. Primary outcomes include: 1) body length and lean body mass by air displacement plethysmography at 36 weeks’ PMA; 2) quantitative magnetic resonance imaging-based measures of brain size and microstructure at term equivalent age; and 3) Bayley-IV scales at 2 years’ corrected age.

**Discussion:**

We expect this trial to provide important data regarding the effectiveness of individually targeted human milk fortification in the neonatal intensive care unit (NICU).

**Trial registration:**

NCT03977259, registered 6 June, 2019.

## Background

For hospitalized very preterm infants, the two to 4 month-long neonatal intensive care unit (NICU) hospitalization coincides with a critical period in development during which the brain is uniquely sensitive to nutrition [1, 2]. The use of human milk is recommended based on strong evidence of short- and long-term benefits [3, 4]. Aiming to support fetal rates of physical growth and tissue accretion [5–7], multi-component human milk fortification is recommended [8, 9] and practiced widely [10]. However, epidemiologic data indicate that NICU growth outcomes remain far short of the goal to match in-utero growth [11, 12]. Additionally, we and others have reported slower weight gain and head circumference growth among fortified human milk-fed as compared with preterm formula-fed infants [13–17], suggesting gaps in current fortification strategies. These gaps are particularly relevant given the recent rise in the use of both maternal and donor milk NICUs [18].

A major challenge in clinical care is that in prescribing nutritional interventions, NICU clinicians rely on published average nutrient values [5, 9] whereas the actual macronutrient content of human milk varies considerably [19]. Pasteurized donor milk is also variable and is, on average, lower in protein content than maternal milk [20, 21]. Thus, over time, and despite standard fortification, some preterm infants may accumulate protein and/or energy deficits that adversely impact their physical growth and/or brain development, with likely implications for long-term neurodevelopmental outcomes.

The recent availability of a reliable point-of-care human milk analyzer provides an opportunity to individually target fortification of human milk in the NICU. However, only a few randomized trials have examined the effectiveness of this approach in improving physical growth [22], and the results have been inconsistent. Additionally, virtually no data exist on outcomes beyond NICU discharge, and effects of an individualized approach to human milk fortification on structural brain development during the neonatal period are unknown.

To address these knowledge gaps, we designed Nourish as a single-center randomized, controlled clinical trial designed with the aim of evaluating the effectiveness of individually targeted human milk fortification, as compared with standard-of-care fortification, in promoting physical growth, structural brain development, and neurodevelopmental outcomes in very preterm infants. Primary outcome domains for Nourish are: 1) physical growth during the intervention; 2) magnetic resonance imaging (MRI) based measures of brain development at term equivalent age; and 3) neurodevelopmental outcomes at 2 years’ corrected age. The overall hypothesis is that minimizing protein and energy deficits by individually targeting human milk fortification during the NICU hospitalization will improve physical growth, regional brain development, and neurodevelopmental function.

## Methods / design

### Setting and eligibility criteria

This randomized clinical trial will take place at Brigham and Women’s Hospital, an academic Level III NICU in Boston, MA. Inclusion criteria are: 1) singleton or twin infant with gestational age at birth 24 0/7 to 30 6/7 weeks; 2) chronologic age < 21 days; and 3) mother providing milk, with clinical consent given to use pasteurized donor human milk if maternal milk is in short supply or unavailable. Exclusion criteria include: 1) major congenital anomaly; 2) birth weight < 3rd percentile [23]; 3) necrotizing enterocolitis or other clinically significant gastrointestinal pathology; 4) clinically significant renal or hepatic dysfunction or inborn error of metabolism; 5) fluid restriction < 140 mL/kg/day anticipated for 3 or more days; 6) grade 3 or 4 intraventricular hemorrhage detected prior to enrollment; and 7) anticipated transfer < 36 weeks’ postmenstrual age or expected death.

### Informed consent

Written, informed consent from one parent will be obtained by a research nurse, dietitian, or physician. Consent documents will be available in English and Spanish and will cover both clinical trial participation and the use of data and/or bio-specimens for future research. Separate consent forms will be used for the NICU and follow-up phases. This study protocol (2019P000893) was approved by the Partners Human Research Committee (FWA00000484) on June 25, 2019.

### Milk collection, pooling, and analysis

As soon as possible after delivery, mothers of all preterm infants admitted to the NICU receive standard written and verbal instructions by a board-certified lactation consultant to express milk at least 8 times per day with a double-electric breast pump, augmented with hand expression to improve emptying. For enrolled infants during the study diet period, each weekday, research assistants will pool fresh maternal milk and supplement with frozen maternal milk and/or donor milk as needed based on the daily volume ordered by the clinical team (typically 150–160 mL/kg/day). From these 24-h pools, 3 mL of milk will be sonicated for 5 s and warmed to 40 °C using a bead warmer, then analyzed for macronutrient content with a mid-infrared spectroscopy-based human milk analyzer (sonicator, bead warmer, and analyzer all Miris AB, Uppsala, Sweden) [24]. Each sample of milk will be analyzed once.

### Intervention

The study period will start the first weekday that the infant reaches the clinical standard goal human milk diet, defined as ≥140 mL/kg/day fortified with multicomponent liquid human milk fortifier (Similac human milk fortifier hydrolyzed protein concentrated liquid, Abbott Laboratories, 5 mL added to 25 mL milk, equivalent to 24 kcal/ounce assuming the ‘base’ milk is 20 kcal/oz) plus liquid protein fortifier (Abbott Laboratories) at 0.27 g/dL (“Step 1” assuming that the ‘base’ milk contains 1 g/dL protein, with the goal to provide ≥4 g/kg/day protein) and is not receiving IV fluids. Throughout the trial, if maternal milk is in short supply or not available, pasteurized donor human milk (“donor milk”) will be used. To reduce variability of donor milk macronutrient composition, particularly of protein which declines with advancing lactation duration [21], we will use pasteurized milk that was expressed within 3 months of delivery, provided by a community-based milk bank affiliated with the Human Milk Banking Association of North America (Mother’s Milk Bank Northeast, Newton MA). Infant formula will not be used for supplementation during the study period. Routine electrolyte monitoring and supplementation and supplementation of Vitamin D and iron for all participants will follow a local Enteral Nutrition Clinical Guideline, available upon request.

**Fig. 1:**
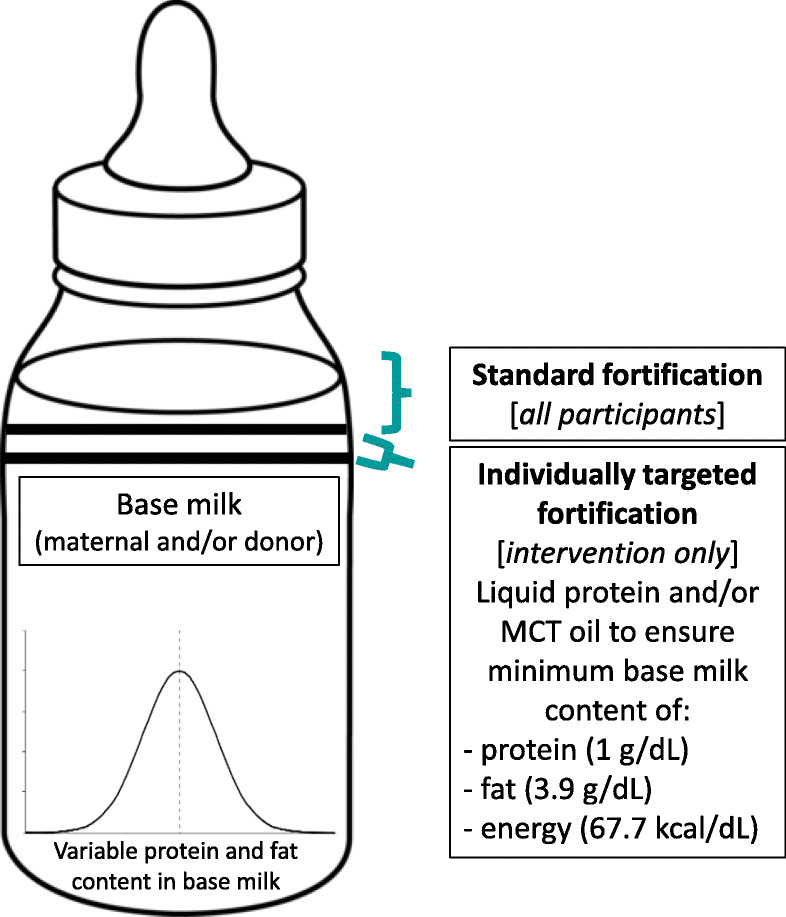
Schematic of standard fortification and individually targeted fortification in the Nourish study. Standard fortification will be provided for all participants and comprises 1) multicomponent liquid human milk fortifier (Similac human milk fortifier hydrolyzed protein concentrated liquid, Abbott Laboratories), 5 mL added to 25 mL milk and 2) liquid protein fortifier (Abbott Laboratories) at 0.27 g/dL. Milk for infants in the intervention group will also be individually fortified with liquid protein (Abbott Laboratories) and/or medium chain triglyceride (MCT) oil (Dot Foods, Nestle USA) to ensure target levels in the ‘base’ milk of 1 g/dL for protein, 3.9 g/dL for fat, and 67.7 kcal/dL for energy. If maternal milk is in short supply, pasteurized donor milk expressed within 3 months of delivery will be used. The study diet will continue through 36 weeks’ postmenstrual age

In addition to the standard-of-care diet of human milk (maternal and/or donor) with multi-component HMF to 24 kcal/oz. and “Step 1” protein, milk for infants in the intervention group will be additionally fortified with liquid protein (Abbott Laboratories) and/or medium chain triglyceride (MCT) oil (Dot Foods, Nestle USA) to ensure target levels in the ‘base’ milk of 1 g/dL for protein and 3.9 g/dL for fat (Fig. 1). Individual recipes are calculated in a custom-built Excel spreadsheet based on data input including the infant’s body weight, daily fluid volume, and milk macronutrients. Currently, we do not have access to a sterile carbohydrate modular, which means that even after target levels of protein and fat are reached, the energy content may remain below the target of 67.7 kcal/dL. Should energy content fall below this target, we will add more MCT oil to achieve the energy target. We will use the following formula to calculate the energy content of milk: 9*fat + 4*protein + 4*carbohydrate [25] with fat, protein, and carbohydrate values taken from the milk analysis. We will use the “true protein” value (crude protein*0.8) to account for non-protein nitrogen [24].

### Management of growth faltering

Growth faltering is defined as an average weight gain < 15 g/kg/day or gain in body length < 0.8 cm per week. Management of growth faltering among participants will proceed as follows: 1) increase enteral feeding volume by 10 mL/kg/day, if acceptable to the clinical team; 2) add MCT oil to increase energy density to 26 kcal/oz. based on assumed ‘base’ milk energy content of 20 kcal/oz.; 3) increase standard liquid protein fortifier to 0.54 g/dL (“Step 2”); and 4) further increase standard energy density to 28 kcal/oz. with MCT oil and/or increase protein to 0.8 g/dL (“Step 3”).

### Criteria for discontinuing or modifying allocated interventions

If growth faltering persists despite taking the above steps, partial formula supplementation will be considered by the clinical and research teams. Participants will be removed from the study if they develop NEC or any other exclusion criteria, or at parent request.

### Outcomes

We will assess physical growth with 1) anthropometry and 2) body composition analysis. At study start and end points, we will weigh infants in duplicate on a calibrated portable digital scale; if the infant is not stable enough to be weighed on a portable scale, we will use the built-in isolette scale. Body length will be measured in duplicate with a recumbent length board and head circumference with a non-stretchable tape and both recorded to 0.1 cm. One of two registered neonatal dietitians will perform all anthropometric measurements, assisted by a trained research assistant and with other clinical assistance (e.g. bedside nurse, respiratory therapist) as indicated. Interrater reliability between the 2 dietitians will be assessed at baseline and quarterly throughout the study period. We will use an infant air displacement plethysmography system (PEA POD, Cosmed, Concord CA) to measure infant body volume and mass and estimate fat mass and fat free mass with a 2-compartment model [26]. Infants will undergo body composition assessment as soon as possible upon study start, with timing based on the ability to remain off respiratory support during the assessment, and again at study end point.

We will assess structural brain development with MRI. Infants will be scanned without sedation at term equivalent age (38–41 weeks’ postmenstrual age, PMA) using a “feed and wrap” method [27]. We will use a 3 Tesla Prisma system (Siemens) equipped with a 16-channel pediatric head coil. To derive the primary volumetric outcomes (total brain volume, cerebellar volume), we will use an automated segmentation method, MANTiS [28]. A secondary MRI outcome will be fractional anisotropy in the posterior limb of the internal capsule (PLIC), a measure of white matter microstructure. We will manually delineate the PLIC using region-of-interest based analysis [29].

To assess neurodevelopment at 2 years’ corrected age, we will use the Bayley Scales of Infant and Toddler Development, 4th edition [30]. Primary outcomes will include the cognitive and motor scores (continuous). Secondary outcomes will include cognitive and motor delay (scores < 85) and the language score (continuous and < 85). We will assess spatial working memory with the “Spin the Pots” task, [31] behavioral inhibition with the “Mommies and Babies” task, [32] and executive function and behavioral difficulties using valid parent questionnaires [33, 34]. At 2 years, we will reassess anthropometry using similar methods to those described previously, and assess blood pressure with an automated oscillometric device.

To promote cohort retention and collect interim data on infant development, we will administer maternal questionnaires at 4 and 12 months’ corrected age, including the Baby Pediatric Symptom Checklist, Motor and Social Development Scale, [35] Ages and Stages, [36] and Infant Toddler Quality of Life scale [37].

### Participant timeline

**Fig. 2:**
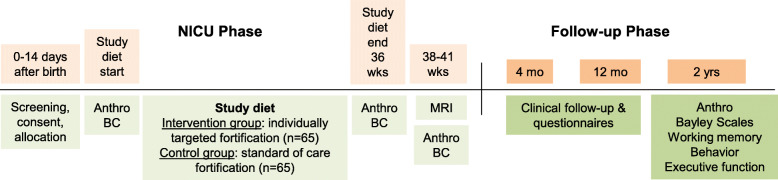
Nourish study timeline of enrollment, interventions, and assessment. The study diet will start the first weekday that the infant reaches a full volume, fortified enteral diet (≥140 mL/kg/day human milk). The study diet will end at 36 weeks’ postmenstrual age (PMA). Anthro is anthropometry (weight, length, head circumference). BC is body composition assessed with air displacement plethysmography. MRI will be obtained at 38–41 weeks’ PMA. Bayley Scales of Infant and Toddler Development, 4th edition will be used. Spatial working memory will be assessed with the “Spin the Pots” task, behavioral inhibition with the “Mommies and Babies” task, and executive function and behavioral difficulties with parent questionnaires (Behavioral Rating Inventory of Executive Function – Preschool Version; and Infant-Toddler Symptom Checklist—Long Version). Ages in the follow-up phase are corrected ages (age corrected for preterm birth)

The timeline of enrollment, interventions, and assessments are shown in Fig. 2.

### Sample size

The planned sample size is 130 infants (65 per study group). Accounting for expected attrition (5% by NICU discharge due to death or unexpected transfer) and an additional 5% loss due to uncorrectable motion artifact for MRI outcomes, we expect to be able to detect a moderate effect of the intervention on primary outcomes (0.49 z-scores in weight, 0.24 z-scores in fat free mass, 24 cc total brain volume) at α = 0.05 and power 0.8. Accounting for 10% further attrition to 2 years’ corrected age, we have adequate power to detect an 8.1-point difference between groups on the Bayley cognitive scale.

### Assignment of interventions

We will use permuted block randomization and assign participants 1:1 to the standard of care diet (control) or individually fortified diet (intervention). Twins will be randomized individually. The study biostatistician will generate the allocation sequence and place the assignments in envelopes, to be opened by the research assistants on the study start date.

### Blinding

Parents, clinical staff, and all study staff apart from the research assistants who prepare the study diet will be blinded to the study group. Blinding will be facilitated by the use of amber enteral feeding tubes to hide the appearance of the milk. Unblinding will be permitted if an error in milk preparation is identified, if a participant experiences growth faltering despite all strategies outlined in the protocol, or if unexpected safety concerns arise that require unblinding.

### Data collection and management

Most study data will be stored in Research Electronic Data Capture (REDCap), a secure, HIPAA compliant application. Maternal questionnaires will be both administered and stored within REDCap. Milk analysis and PEAPOD data will be downloaded directly from the devices onto flash drives and transferred to a secure shared server. MRI files will be transferred directly from the scanner to a research server.

### Collection and storage of biological specimens for future use

We will collect 2 mL of unfortified milk and 1 mL of fortified milk each time milk is prepared for the study diet and store it at − 80 °C.

### Statistical methods

Our overall analytic strategy compares pre-specified primary and secondary outcomes between control and intervention groups. We will follow the intention-to-treat principle and analyze data for all enrolled participants. We will use multiple imputation to address missing data due to death or loss to follow-up. Results will be expressed as mean or median differences with 95% confidence intervals. We will use mixed linear models adjusted for intrafamilial clustering due to multiple births, along with control for baseline covariates, as needed, to minimize effects of between-group differences in baseline factors due to chance. We will examine intervention effects in the complete cohort and use stratification approaches to explore the effects of variables specified a priori including sex, fetal growth status (SGA vs. non-SGA), and gestational age (24–27 weeks vs. 28–30 weeks).

## Discussion

Here, we describe the protocol for a randomized, controlled trial of individually targeted human milk fortification during the NICU hospitalization for preterm infants born at 24 to 30 completed weeks’ gestation. We will measure the effectiveness of this intervention in terms of 1) increasing physical growth and optimizing body composition during the NICU hospitalization; 2) improving structural brain development by term equivalent age; and 3) improving neurodevelopmental outcomes at 2 years’ corrected age.

Our protocol has several strengths. One is the integration of our intervention into the NICU workflow. Another is our combination of standard and innovative outcome measures. For example, we will assess not just standard anthropometry but also infant body composition, including fat-free mass which indexes muscle and organ growth. Brain MRI at term equivalent age will enable us to detect short-term effects of the nutritional intervention on brain size and microstructure. At 2 years’ corrected age, measures of working memory and behavioral inhibition, which reflect emerging executive function, as well as measures of sustained attention and self-regulation will complement the standard Bayley scales. Another strength is the “triple blinding” of parents, clinicians, and study staff to minimize biases that could affect participant allocation, assessment, and/or adherence to the intervention. Limitations include the single-center design, which may reduce generalizability, and the sample size, which may preclude the detection of small but clinically important effects. Additionally, the intervention is labor intensive, with future work needed to optimize for scalability.

In conclusion, we have designed a randomized controlled trial to test the effectiveness of individually targeted human milk fortification during the NICU hospitalization. This nutritional intervention during a critical period in preterm infant brain development has the potential to improve both short and long-term outcomes for very preterm infants.

## Data Availability

N/A

## Abbreviations

PMA: Postmenstrual age
NICU: Neonatal intensive care unit
MCT: Medium chain triglyceride
PLIC: Posterior limb of the internal capsule
MRI: Magnetic resonance imaging
SGA: Small for gestational age
